# A new species of *Pelodiscus* from northeastern Indochina (Testudines, Trionychidae)

**DOI:** 10.3897/zookeys.824.31376

**Published:** 2019-02-13

**Authors:** Balázs Farkas, Thomas Ziegler, Cuong The Pham, An Vinh Ong, Uwe Fritz

**Affiliations:** 1 21 Bercsényi St., 2464 Gyúró, Hungary Unaffiliated Gyúró Hungary; 2 Cologne Zoo, 173 Riehler St., 50735 Cologne, Germany Cologne Zoo Cologne Germany; 3 Institute of Zoology, Cologne University, 47b Zülpicher St., 50674 Cologne, Germany Cologne University Cologne Germany; 4 Institute of Ecology and Biological Resources, Vietnam Academy of Science and Technology, 18 Hoang Quoc Viet St., Cau Giay, Hanoi, Vietnam Institute of Ecology and Biological Resources, Vietnam Academy of Science and Technology Hanoi Vietnam; 5 Department of Zoology, Vinh University, 182 Le Duan St., Vinh City, Nghe An Province, Vietnam Vinh University Vinh Vietnam; 6 Museum of Zoology, Senckenberg Dresden, A. B. Meyer Building, 01109 Dresden, Germany Museum of Zoology, Senckenberg Dresden Dresden Germany

**Keywords:** China, genetics, morphology, softshell turtles, Vietnam

## Abstract

A new, critically endangered species of softshell turtle, *Pelodiscusvariegatus***sp. n.** is described from north-central Vietnam and Hainan Island, China, distinguished by a unique set of genetic and morphological traits from all other congeners (*P.axenaria*, *P.maackii*, *P.parviformis*, *P.sinensis*, and unnamed genetic lineages). Morphologically, *P.variegatus* is characterized, among others, by its strong ventral ornamentation in all age classes.

## Introduction

“Chinese softshell turtles” were considered for decades to represent the morphologically highly variable and geographically widespread species *Pelodiscussinensis* (Wiegmann, 1834), distributed from the Russian Far East through the Korean Peninsula, eastern and central China to Vietnam (e.g., Pope 1935; Wermuth and Mertens 1961, 1977; Pritchard 1979; Meylan 1987; Ernst and Barbour 1989). Another species, *P.maackii* (Brandt, 1857), from the northernmost part of the distributional range, was resurrected from the synonymy of *P.sinensis* by Chkhikvadze (1987) employing osteological features. On the basis of morphological characters, two additional species from central China were described in the 1990s, *P.axenaria* (Zhou, Zhang & Fang, 1991) and *P.parviformis* Tang, 1997. However, the validity of the latter three species was repeatedly questioned or rejected (Ernst et al. 2000; TTWG 2007). Using three mitochondrial DNA (mtDNA) fragments and one nuclear locus, Fritz et al. (2010) confirmed that *Pelodiscus* represents a species complex. These authors tentatively recognized *P.axenaria* and *P.maackii* and highlighted that the assignment of scientific names was difficult because the identity of the oldest available name, *Trionyxsinensis* Wiegmann, 1834, remained unclear. Stuckas and Fritz (2011) designated a lectotype for this species and succeeded in sequencing approximately 1500 base pairs of mtDNA of this 180-year-old type specimen. This allowed the conclusive recognition of four genetically distinct species, *P.axenaria*, *P.maackii*, *P.parviformis*, and *P.sinensis*. Yang et al. (2011) arrived at the same judgment with respect to *P.parviformis* after evaluating molecular and morphological data but emphasized the near-impossibility of distinguishing *P.axenaria* from *P.parviformis* by external characters alone. Finally, based on comprehensive sampling, Gong et al. (2018) examined the diversity of *Pelodiscus* using mtDNA and five nuclear loci. These authors showed that the diversity of *Pelodiscus* is still underestimated and provided evidence that two genetically and morphologically distinct *Pelodiscus* species occur syntopically in northern Vietnam. One of these species was found widely distributed and occurring also in China, whereas the other seemed to be confined to northern and central Vietnam. Mitochondrial DNA of the two species was only moderately divergent, albeit representing distinct lineages. In contrast, the studied nuclear loci revealed discrete gene pools, suggestive of ancient mitochondrial capture. In the present study, we describe this as yet unnamed taxon with introgressed mitochondria.

## Taxonomy

### Pelodiscus variegatus sp. n.

Taxon classificationAnimaliaTestudinesTrionychidae

http://zoobank.org/95F21749-6C4A-439F-BBC0-A44B6F82F1AA

**Figure 1.**
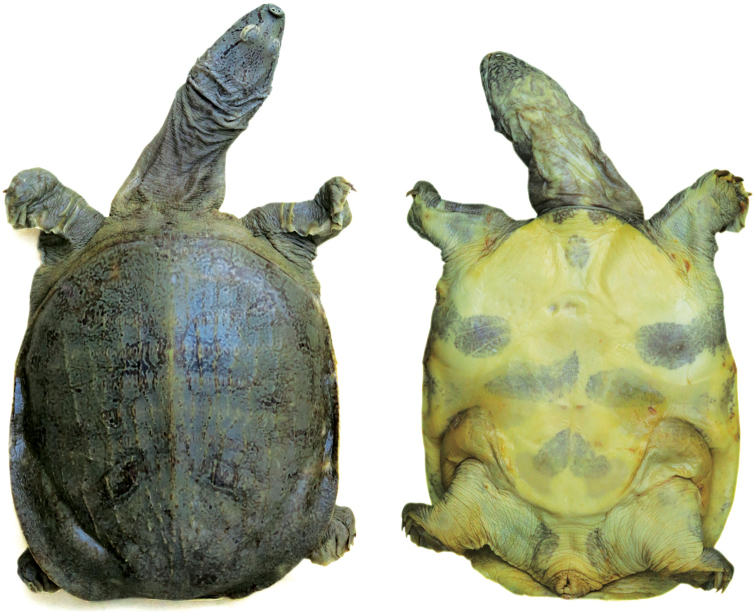
Dorsal and ventral aspects of the holotype of *Pelodiscusvariegatus* sp. n. (IEBR 4480, adult female, 134.2 mm PL). Photographs Balázs Buzás.

**Figure 2.**
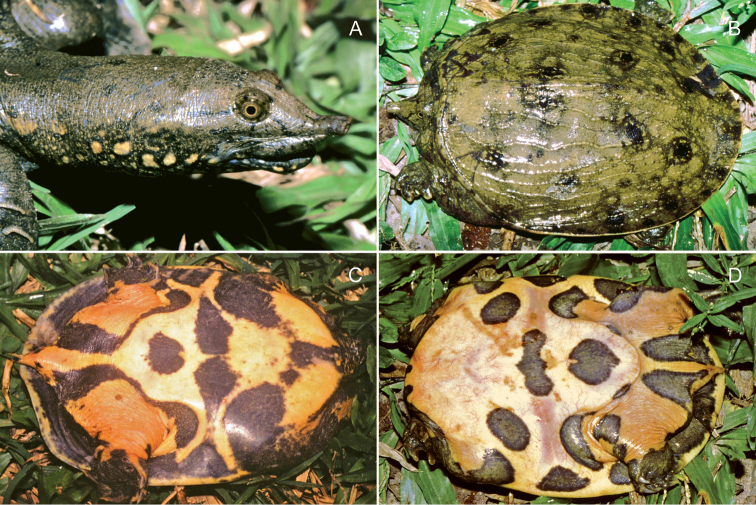
Two paratypes of *Pelodiscusvariegatus* sp. n. in life. **A, C** MTD 44045, female, 75.2 mm PL**B, D** MTD 42834, female, 86.6 mm PL. Photographs Thomas Ziegler.

**Figure 3.**
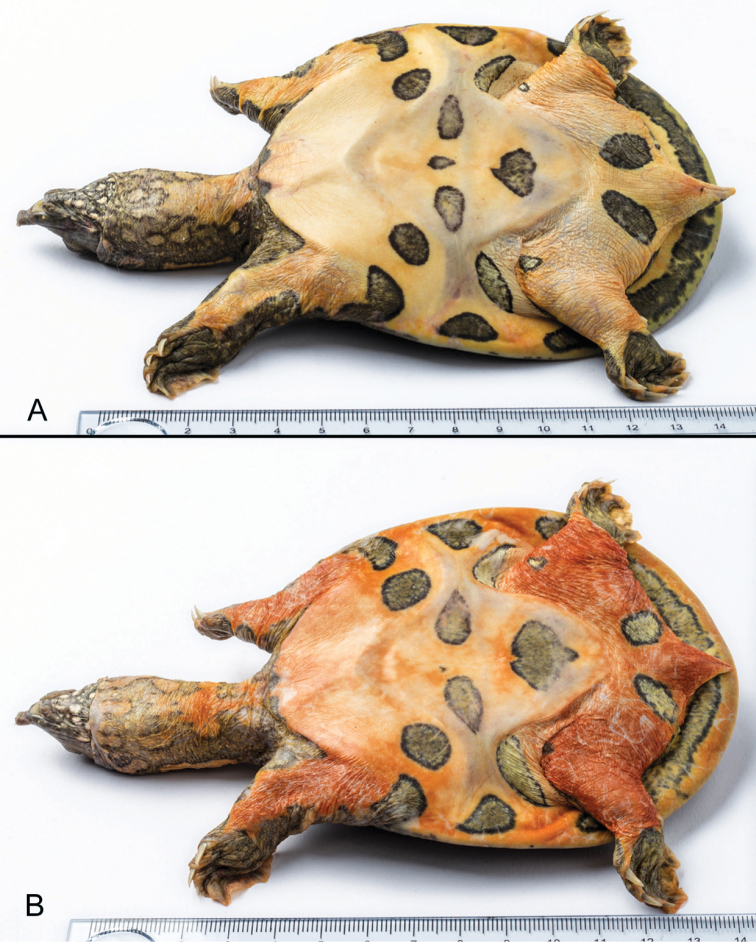
Plastral views of two freshly dead paratypes of *Pelodiscusvariegatus* sp. n. **A** HNHM 2018.112.1, female, 77.4 mm PL**B** AQT001-HTVN2018, female, 77.3 mm PL. Photographs An Vinh Ong.

**Figure 4.**
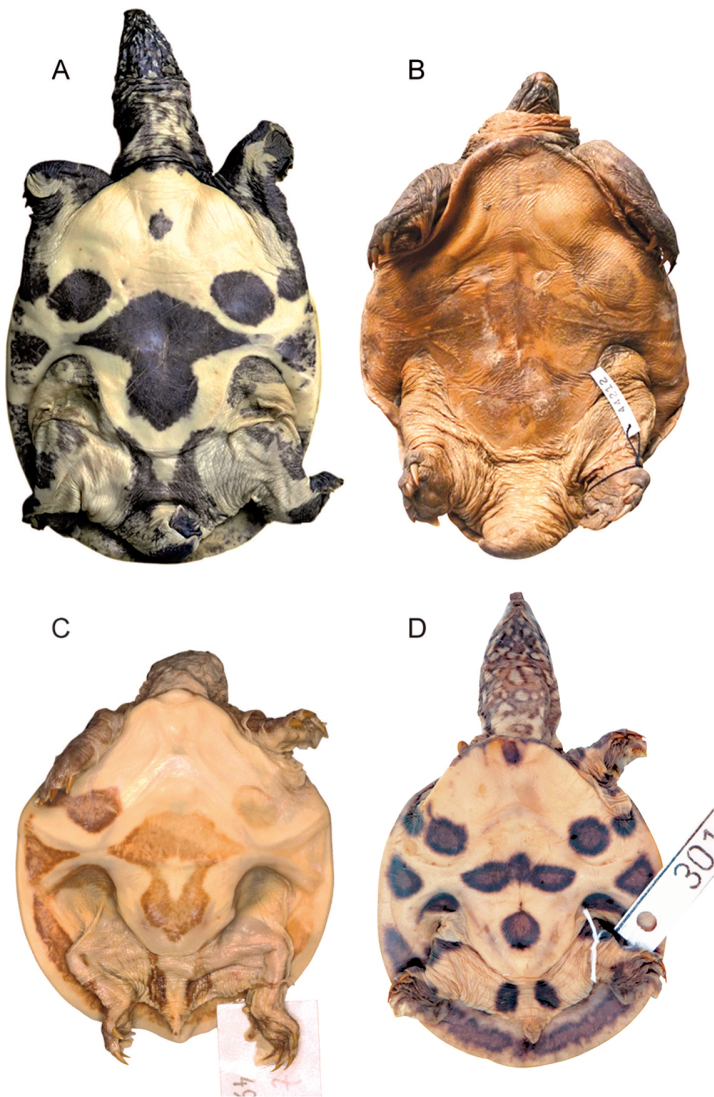
Variation in plastral ornamentation of *Pelodiscusvariegatus* sp. n. **A** HNHM 2018.111.1, adult male, 109.7 mm PL**B** ZFMK 44212, adult male, 114.4 mm PL**C** juvenile, ZMB 49776, 50.0 mm PL**D** hatchling, AMNH 30125, 37.2 mm PL. Not to scale. Photographs Balázs Buzás (**A**), Balázs Farkas (**B**), Frank Tillack (**C**), Lauren Vonnahme (**D**).

Figs 1 , 2 , 3 , 4 , Tables 1 , 2 , 3 , 4

#### Holotype.

Institute of Ecology and Biological Resources, Hanoi: IEBR 4480, adult female preserved in alcohol, Thai Thinh village, Kinh Mon District, Hai Duong Province, Vietnam, leg. Cuong The Pham, 20 June 2009 (Fig. 1).

#### Paratypes

(all preserved in alcohol). American Museum of Natural History, New York: AMNH 30125, hatchling, Nodoa (= Nada, Danzhou), Hainan Province, China, leg. Clifford H Pope, December 1922–July 1923; Hungarian Natural History Museum, Budapest: HNHM 2018.111.1, adult male, same data as for the holotype; HNHM 2018.112.1, female, Song Rac Lake, Cam Xuyen District, Cam Lac Commune, Ha Tinh Province, Vietnam (18.1665N, 106.0957E), leg. An Vinh Ong, Quang Xuan Hoang and Trung Van Vo, 20 October 2018; Museum of Zoology, Senckenberg Dresden: MTD 42534, adult female, through local trade (Nha Trang), Khanh Hoa Province, Vietnam, leg. Edgar Lehr, February 2000; MTD 42834, female, through local trade (from lowland forest northwest of Ky Thuong village; Ziegler 2002), Ha Tinh Province, Vietnam, leg. Thomas Ziegler, 12 August 1997 (field number TZ 584); MTD 44045, female, through local trade (allegedly captured in a sandy stream in Phong Nha–Ke Bang Reserve; Ziegler et al. 2004), Quang Binh Province, Vietnam, leg. Thomas Ziegler, August/September 2001 (field number TZ V8); Natural History Museum Vienna: NMW 30221:1–6, six hatchlings, Phuc Son, Tan Yen District, Bac Giang Province, Vietnam, leg. Hans Fruhstorfer, 1903; Zoological Collection, Vinh University, Vinh, Nghe An Province: AQT001-HTVN2018, female, Song Rac Lake, Cam Xuyen District, Cam Lac Commune, Ha Tinh Province, Vietnam (18.1665N, 106.0957E), leg. An Vinh Ong, Quang Xuan Hoang and Trung Van Vo, 20 October 2018; Zoological Research Museum Alexander Koenig, Bonn: ZFMK 101820, female, through local trade (surroundings of the area named “Chin Xai” by local people; Ziegler 2002), Ha Tinh Province, Vietnam, leg. Thomas Ziegler, 23 August 1997 (field number TZ 679).

#### Additional specimens

(all preserved in alcohol). American Museum of Natural History, New York: AMNH 28345, adult female, Nodoa (= Nada, Danzhou), Hainan Province, China, leg. Clifford H Pope, December 1922–July 1923; Field Museum, Chicago: FMNH 6626 and 6627, females, Nodoa (= Nada), Hainan Province, China, leg. Clifford H Pope, 1923; Museum of Vertebrate Zoology, Berkeley: MVZ 23946, male, Kachek (= Jiaji, Qionghai), Hainan Province, China, 20 m a.s.l. (23.36667N, 116.65E), leg. J Linsley Gressitt, 8 August 1935; Natural History Museum Vienna: NMW 30219:1, female, River of Mount Wuchi (= Wanquan He, Wuzhi Shan), Hainan Province, China, don. Franz Steindachner, 1906; NMW 30232:3, adult male, Kau-kong River (to be identified with Gaogong He according to Zhao and Adler 1993), Hainan Province, China, don. Franz Steindachner, 1906; Naturalis Leiden: RMNH 4752 and 4753, juveniles, “Annam” (= possibly Phuc Son, Tan Yen District, Bac Giang Province, Vietnam, the declared origin of all of Fruhstorfer’s specimens in other collections), leg. Hans Fruhstorfer, 1903; Zoological Research Museum Alexander Koenig, Bonn: ZFMK 44212 and 44213, adult males, near Hanoi, Vietnam, leg. Ivan Rehák, March 1984; ZFMK 44214, adult female, near Hanoi, Vietnam, leg. Ivan Rehák, March 1984; ZFMK 59199 and 59200, females, Red River (Song Hong), Hanoi, Vietnam, leg. Václav Laňka, June 1985; Natural History Museum Berlin: ZMB 29614, 49775 and 49776, juveniles, Phuc Son, Tan Yen District, Bac Giang Province, Vietnam, leg. Hans Fruhstorfer.

#### Diagnosis.

In the 12S rRNA gene, *Pelodiscusvariegatus* differs from all other species and genetic lineages of *Pelodiscus* by the presence of cytosine (C) instead of thymine (T) at position 96 of the reference alignment (Suppl. material 1). In the cyt *b* gene, *P.variegatus* differs from all other species and genetic lineages of *Pelodiscus* by the presence of adenine (A) instead of cytosine (C) in position 130 and by the presence of thymine (T) instead of cytosine (C) in positions 204, 741, and 1081 of the reference alignment (Suppl. material 2). In the mtDNA fragment corresponding to the partial ND4 gene plus adjacent DNA coding for tRNAs, *P.variegatus* differs from all other species and genetic lineages of *Pelodiscus* by the presence of adenine (A) instead of guanine (G) in position 94 of the reference alignment (Suppl. material 3). These and further species-specific differences are shown in Tables 1–3.

**Table 1.** Selection of diagnostic sites of the 12S rRNA gene for *Pelodiscus* species (84 wild-caught individuals from Gong et al. 2018). Positions refer to the 400-bp-long reference alignment in the Supporting Information. G, H, and I are genetic lineages that represent putatively distinct taxa (Gong et al. 2018).

	*n*	13	14	20	34	96	222	223	224	225	234	327	330	384	385
*P.variegatus* sp. n.	4	T	T	T	T	C	T	T	T	T	A	C	T	G	C
* P. axenaria *	5	.	.	A	C	T	C	.	C	A	C	T	C	C	T
G	1	.	.	.	.	T	.	.	.	.	.	.	.	.	.
H	1	.	.	.	.	T	.	.	.	.	.	.	.	.	.
I	6	.	.	.	C	T	C	.	C	A	C	.	.	C	.
* P. maackii *	10	.	.	C	.	T	.	G	.	.	.	.	.	.	.
* P. parviformis *	8	C	C	C	C	T	.	G	.	.	.	.	.	.	.
* P. sinensis *	49	.	.	.	.	T	.	.	.	.	.	.	.	A/.	.

**Table 2.** Selection of diagnostic sites of the cyt *b* gene for *Pelodiscus* species (94 wild-caught individuals from Gong et al. 2018). Positions refer to the 1168-bp-long reference alignment in the Supporting Information. G, H, and I are genetic lineages that represent putatively distinct taxa (Gong et al. 2018).

	*n*	130	132	147	148	195	204	285	288	315	477	520	730	741	1081
*P.variegatus* sp. n.	4	A	A	C	C	G	T	T	C	C	T	T	A	T	T
* P. axenaria *	10	C	C	T	T	A	C	.	T	.	.	C	G	C	C
G	1	C	C	.	.	.	C	C	.	T	.	C	G	C	C
H	1	C	C	.	.	.	C	C	.	T	.	C	G	C	C
I	6	C	C	.	T	A	C	.	T	.	.	C	G	C	C
* P. maackii *	11	C	C		.	A	C	C	.	.	.	C	G	C	C
* P. parviformis *	10	C	C	.	.	A	C	C	.	T	C	C	G	C	C
* P. sinensis *	51	C	.	.	.	.	C	C	.	T	.	C*	G*	C	C

*Among 51 sequences of *P.sinensis*, only the holotype had the same character state as *P.variegatus*.

**Table 3.** Selection of diagnostic sites of the mtDNA fragment comprising the partial ND4 gene and adjacent DNA coding for tRNAs for *Pelodiscus* species (91 wild-caught individuals from Gong et al. 2018). Positions refer to the 838-bp-long reference alignment in the Supporting Information. G, H, and I are genetic lineages that represent putatively distinct taxa (Gong et al. 2018).

	*n*	10	18	31	64	94	148	151	211	262	263	301	305	508
*P.variegatus* sp. n.	4	C	T	C	A	G	A	G	G	C	C	C	T	A
* P. axenaria *	10	.	.	T	G	A	C	A	A	T	T	T	C	G
G	1	.	.	.	.	A	.	.	A	.	.	.	.	G
H	1	.	.	.	.	A	.	.	A	.	.	.	.	G
I	6	T	C	T	.	A	C	A	A	T	.	.	A	G
* P. maackii *	10	.	.	.	.	A	.	.	A	.	.	.	.	G
* P. parviformis *	9	.	.	.	.	A	G	A	A	.	.	.	.	G**
* P. sinensis *	50	.	.	.	.	A	G*	.	A/.	.	.	.	.	G**

*Among 50 *P.sinensis*, only one individual had the same character state as *P.variegatus*. **Among nine *P.parviformis* and 50 *P.sinensis*, two and one individuals, respectively, had the same character state as *P.variegatus*.

In addition to the genetic distinctiveness of *P.variegatus*, the strong ventral ornamentation clearly sets apart adult individuals from *P.maackii*, which has a uniform yellowish white or straw yellow plastron devoid of any markings in adults; from *P.sinensis*, which may retain faint remnants of its juvenile pattern on its snow white to reddish white plastron but the round to oval spots are usually isolated and proportionally much smaller; from *P.axenaria*, which has a yellowish white plastron with just a single large black central figure enclosed by the hypo- and xiphiplastra throughout its life (Zhou et al. 1991); and from *P.parviformis*, which has an unmarked yellowish white plastron at all ages. According to the specimens investigated and data available to us, *P.variegatus* also reaches a much smaller maximum size (23 cm CL; AMNH 28345) than *P.maackii* (at least 35 cm CL; Brandt 1857) but grows bigger than *P.parviformis* (16 cm CL; NMW 30232:6); *P.sinensis* (23 cm CL; ZMB 9784) and *P.axenaria* (20 cm CL; Gong et al. 2018) attain dimensions resembling *P.variegatus*. The diagnostic morphological features of adults of these five *Pelodiscus* species are summarized in Table 4.

**Table 4.** Diagnostic morphological features of adults of *Pelodiscus* species.

	* P. axenaria *	* P. maackii *	* P. parviformis *	* P. sinensis *	*P.variegatus* sp. n.
Maximum carapace length (in cm)	20	more than 35	16	23	23
Prevalent carapace color	yellowish brown	olive brown to dark brown	yellowish brown	olive green to olive gray	yellowish brown
Carapace pattern	blurred dark mottling with indistinct stellate spots and ill-defined half oval blotches around perimeter of leathery margin	fine dark-edged yellowish to orange spots, background sometimes dark-mottled	dark marbling with indistinct stellate spots and ill-defined half oval blotches around perimeter of leathery margin	none or small, irregular black blotches and vermiculations or small, faint stellate spots	complex dark marbling, large, irregularly disposed black stellate spots and half oval blotches around perimeter of leathery margin
Prevalent plastron color	yellowish white	white to straw yellow	yellowish white	snow white to pinkish white	pinkish white to pale reddish orange
Plastral pattern	a single dark gray central figure enclosed by hypo- and xiphiplastra, underside of leathery margin of carapace unmarked	no pattern, underside of leathery margin of carapace unmarked	no pattern, underside of leathery margin of carapace unmarked	no pattern or relatively small, faint round to oval dark markings, underside of leathery margin of carapace unmarked	distinct, large dark blotches, underside of leathery margin of carapace pigmented
Head and neck pattern	numerous fine dark brown to black markings, pre- and postocular stripes thin and discontinuous	fine dark-edged yellowish to orange spots, pre- and postocular stripes thick, edged in yellowish white	numerous fine dark brown to black markings, pre- and postocular stripes thin and discontinuous	a few scattered dark and light markings, pre- and postocular stripes of medium thickness, sometimes accentuated with white	numerous fine dark brown to black markings edged in yellowish white, pre- and postocular stripes thick
Throat pattern	minuscule, indistinct yellowish white spots	large light, dark-edged markings	minuscule, barely discernible black spots	small whitish spots or large light, dark-edged markings	large light, dark-edged markings
Carapace tuberculation	dorsal tubercles in longitudinal series more or less discrete, central tubercle in front of marginal ridge of carapace small	tubercles restricted to leathery margin, central tubercle in front of marginal ridge of carapace distinct	dorsal tubercles in longitudinal series more or less discrete, central tubercle in front of marginal ridge of carapace small	dorsal tubercles in longitudinal series more or less discrete, central tubercle in front of marginal ridge of carapace small	dorsal tubercles more or less fused with one another in longitudinal series, central tubercle in front of marginal ridge of carapace indistinct
Medial keel	high	low, carapace flat or longitudinally depressed in middle	high	low, carapace evenly arched or longitudinally depressed in middle	high

#### Description of the holotype.

Carapace length (CL) 171.0 mm, carapace width (CW) 148.0 mm, plastron length (PL) 134.2 mm, head width (HW) 32.2 mm, eye diameter 9.8 mm, interorbital distance 5.4 mm, snout length (SL) 13.3 mm. Carapace oval, slightly domed but with a medial keel, widest at level of the posterior buttress spurs of the hypoplastra. Marginal ridge low, central tubercle indistinct. Dorsal surface roughened by longitudinal ridging and smaller protuberances spread over the leathery margin. The yellowish gray carapace is adorned with an extremely complex greenish black pattern consisting of reticulations and stellate spots enclosed by incomplete rings of the same color, finely dotted with siskin green on either side of the vertebral keel, with those above the pelvis being more pronounced but with additional ones towards the perimeter of the bony disk. The ridging of the carapace is enhanced by the siskin green color of the vertebral keel and the longitudinal rows of tubercles.

Ventral surfaces yellowish white with distinct greenish gray blotches extending onto the plastron. Two dark patches below the neck along the anterior carapace margin, one oval mark between the epiplastra, one on both sides behind the armpits and continuing towards but not reaching the hyoplastra, as well as on the hyo- and hypoplastra and the xiphiplastra, the latter meeting along the midline but not contacting those covering the hyo- and hypoplastra. Additional blotches at the insertion of the hindlimbs and some vague, bruise-like markings on the bridge and the underside of the leathery margin.

Head, extended to posterior level of eyes, terminating in flexible snout. Jaws closed, each covered by fleshy lips except anteriorly where the horny beaks are exposed. Top of head with fine, greenish black specks and streaks. Pre-, sub- and postocular stripes thick (approx. 2 mm wide), locally interrupted and yellowish black in color with thin siskin green outlines. Chin with a contrasting yellowish white pattern on yellowish gray ground, which gradually fades towards the throat and gets almost indiscernible at the base of the neck.

Fore- and hindfeet well-webbed, having five digits each, with claws on the first three digits only. Each forelimb with four antebranchial scales, three of them free-edged. These are wide, the upper one stretching across nearly the whole width of the forelimb (approx. 16 mm long) and the lower two overlapping each other (approx. 20 mm together). Each hindlimb with two horny scales, one smooth on the posterodorsal surface while the other, which is free-edged, is located on the posteroventral surface.

Tail short, barely extending beyond the rear margin of the carapace.

Undersurface of soft parts of body yellowish white embellished with large yellowish gray markings, encroaching on soles and palms, and on either side of the tail.

#### Variations.

There is considerable, in part also age-dependent, variation in pattern intensity among *Pelodiscusvariegatus*. For instance, our male paratype (HNHM 2018.111.1; 109.7 mm PL; Fig. 4A) has somewhat smaller but much more conspicuous stellate spots on its carapace and very large dark (greenish black) markings on its undersurfaces. Those on the hyo-, hypo- and xiphiplastra are fused into a single mushroom-shaped figure, while the “bruises” on the bridge and the ventral surface of the leathery margin also manifest themselves as true blotches.

In one of our female paratypes (MTD 44045; 75.2 mm PL; Fig. 2A, C) the dark blotches on either side of the tail are connected with those at the insertion of the hindlimbs, the leathery margin and the bridge, whereas the ones in front of the entoplastron are fused with those in the armpits, extend towards the central patch between the epiplastra and actually reach the marks on the anterior edge of the plastron, at the base of the neck.

In a battered, presumably very old male examined by us (ZFMK 44212; 116.4 mm PL; Fig. 4B) comparable in size to our male paratype, the ventral ornamentation has lost definition but is still perceptible.

Hatchlings (Fig. 4C, D) have similar markings to adults but the overall effect is even more striking. The parts colored yellowish white in preservative are orpiment to reddish orange in life (Farkas and McCormack 2010) and fade remarkably slowly as age advances (Figs 2–3). In some individuals the light blotches framed with black on the sides of the neck intermingle to form wide bands on a yellowish gray (in life yellowish brown) ground (Fig. 2A). The subocular stripes are occasionally reduced to short streaks and vary between two and three in number. Although the dark longitudinal striation on the nape of juveniles dissolves in adults, a central spot typically remains discernible just in front of the marginal ridge.

Our modest sample size does not allow us to draw definite conclusions about ontogenetic variation and it is presently unknown at what CL sexual maturity is reached in this species. Individuals are sexually clearly dimorphic, with males having much longer and thicker tails, at a PL of 98.8 mm (ZFMK 44213) but a slight variation in TL can be noticed even among hatchlings (NMW 30221:1–6). Anyhow, smaller (younger) specimens appear to possess proportionally wider heads and rounder shells than larger (older) ones. PL/HW 3.50–4.44, mean 3.925; CL/CW 1.02–1.23, mean 1.149; HW/SL 1.98–2.60, mean 2.213, CL/PL 1.18–1.43, mean 1.283.

#### Distribution.

**Figure 5.**
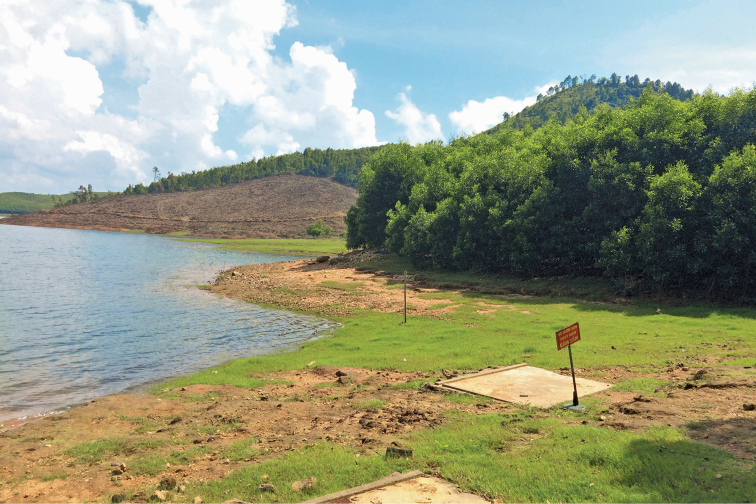
Habitat of *Pelodiscusvariegatus* sp. n.: Song Rac Lake, Cam Xuyen District, Ha Tinh Province, Vietnam. Photograph An Vinh Ong.

**Figure 6.**
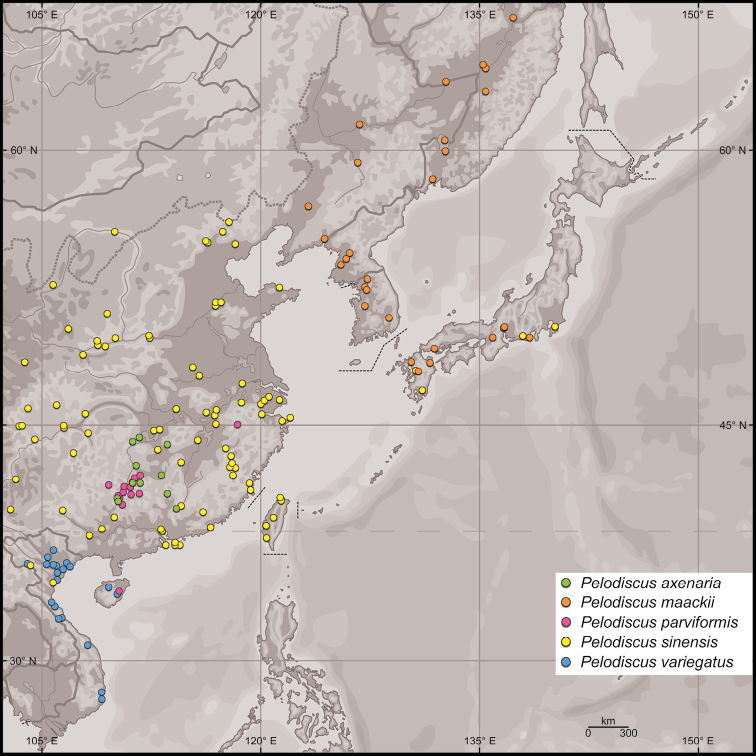
Currently known presence points of *Pelodiscus* species based on our own data as well as distribution maps published by the TTWG (2017) and Gong et al. (2018). Earlier records of *P.sinensis* from Hainan Island are referable to *P.parviformis* or *P.variegatus* sp. n. (see Remarks).

The exact range is unknown but includes lowland areas in the provinces Bac Giang, Ha Tinh (Fig. 5), Hai Duong (own data), “Hai Hung” (a former administrative unit encompassing present-day Hai Duong and Hung Yen; Rudolphi and Weser 1998), Ninh Binh (Rudolphi and Weser 1998; Nguyen et al. 2009; Farkas and McCormack 2010), Phu Tho (Nguyen et al. 2009), Quang Binh (own data), Quang Nam (Farkas and McCormack 2010), Tuyen Quang (Rudolphi and Weser 1998) and Yen Bai (Nguyen et al. 2009) of Vietnam as well as the lower reaches of the Gaogong and Wanquan rivers in Hainan Province, China (own data; Fig. 6). In northeastern Vietnam the distribution area of *Pelodiscusvariegatus* overlaps with that of *Paleasteindachneri* (Bac Giang Province, NMW 23395, NMW 23480:2, Siebenrock 1906; Ha Tinh Province, ZFMK 81539, ZFMK 81540, Ziegler 2002; Quang Binh Province, Ziegler et al. 2004) and further south (Gong et al. 2018) with that of *Pelodiscussinensis*, believed to have been introduced (Nguyen et al. 2009). On Hainan, *Pelodiscusvariegatus* seems to be sympatric with *Pelodiscusparviformis* (vouchers NMW 30232:1–2, NMW 30232:4–8; see Remarks) and *Paleasteindachneri* (NMW 20373; Siebenrock 1906). In Vietnam, most records fall within the “Northeast Lowlands Subregion” of Bain and Hurley (2011; encompassing the “Red River System” of Kottelat 1989). The zoogeographical affinities of Hainan lie also with this area (as well as mainland southwestern China), while the southern portion of the purported range forms part of the “Central–South Vietnam Lowlands Subregion” of Bain and Hurley (2011) or the “Annam River System” of Kottelat (1989).

#### Etymology.

The specific epithet *variegatus* (spotted) is a Latin adjective in masculine gender alluding to the highly contrasting markings, especially the large plastral blotches, of the new species.

#### Remarks.

In addition to the characters used here for diagnosing *P.variegatus*, Gong et al. (2018) described some further genetic differences to other *Pelodiscus* species.

Fritz et al. (2010) suggested that the taxon now named *Pelodiscusvariegatus* resembles *P.parviformis*, prompting the TTWG (2011, 2012, 2014, 2017) to identify the *Pelodiscus* records from Vietnam with the latter species. However, as explained in Gong et al. (2018), this is no longer tenable in the face of the genetic distinctness of the two species.

Traditionally, Chinese softshell turtles from Hainan were identified as *P.sinensis* (e.g., Pope 1935; Ernst and Barbour 1989; Ernst et al. 2000; TTWG 2011, 2012, 2014, 2017). However, the few old (early 20^th^ century) museum specimens serving as record sources represent either *P.variegatus* (AMNH 28345, AMNH 30125, FMNH 6626, FMNH 6627, MVZ 23946, NMW 30219:1, NMW 30232:3) or *P.parviformis* (NMW 30232:1–2, NMW 30232:4–8). Thus, the native occurrence of *P.sinensis* sensu stricto on Hainan seems questionable, even though this species is now most likely bred there in local farms. We cannot exclude that also some of the presence points of *P.sinensis* from southwestern mainland China mapped by the TTWG (2017) refer to *P.parviformis* or *P.variegatus* (and in part perhaps to *P.axenaria*).

#### Conservation implications.

While *Pelodiscussinensis* is listed as “Vulnerable (VU)” by the IUCN Red List of Threatened Species (Asian Turtle Trade Working Group 2000), the conservation status of *P.axenaria*, *P.maackii*, *P.parviformis*, and now *P.variegatus*, remains unassessed, in spite of their proven genetic distinctness (Fritz et al. 2010; Yang et al. 2011; Gong et al. 2018). Given their restricted distributional ranges and the intense exploitation to which they are subjected, all these species would certainly classify for a higher category rating. In this vein, the most recent red list of Chinese vertebrates compiled by Jiang et al. (2016) proposed the conservation status of *P.axenaria*, *P.parviformis* and *P.sinensis* be upgraded to “Endangered (EN)” and indicated *P.maackii* to be “Data Deficient (DD).” Rhodin et al. (2018) suggested for *P.parviformis* “Critically Endangered (CR)” and for *P.sinensis* “Endangered (EN),” whereas *P.axenaria* and *P.maackii* were identified as “Data Deficient (DD).” Consequently, also *P.variegatus*, which was included in *P.parviformis* by Rhodin et al. (2018), should be classified as “Critically Endangered (CR).”

## Supplementary Material

XML Treatment for Pelodiscus variegatus

## Supplementary materials

Supplementary material 1 Reference alignment of 397 bp of the 12S rRNA gene of different *Pelodiscus* species and lineages Data type: molecular data This is an open access article distributed under the terms of the Creative Commons Attribution License (CC BY 4.0), which permits unrestricted use, distribution, and reproduction in any medium, provided the original author and source are credited.Balázs Farkas, Thomas Ziegler, Cuong The Pham, An Vinh Ong, Uwe Fritz

Supplementary material 2 Reference alignment of the cyt b gene of different *Pelodiscus* species and lineages (1168 bp) Data type: molecular data This is an open access article distributed under the terms of the Creative Commons Attribution License (CC BY 4.0), which permits unrestricted use, distribution, and reproduction in any medium, provided the original author and source are credited.Balázs Farkas, Thomas Ziegler, Cuong The Pham, An Vinh Ong, Uwe Fritz

Supplementary material 3 Reference alignment of the partial ND4 gene and adjacent DNA coding for tRNAs (838 bp) of different *Pelodiscus* species and lineages Data type: molecular data This is an open access article distributed under the terms of the Creative Commons Attribution License (CC BY 4.0), which permits unrestricted use, distribution, and reproduction in any medium, provided the original author and source are credited.Balázs Farkas, Thomas Ziegler, Cuong The Pham, An Vinh Ong, Uwe Fritz
